# Grief after the death of a close relative: a study of associated factors and perceptions of bereavement support

**DOI:** 10.1186/s12904-026-02140-x

**Published:** 2026-05-13

**Authors:** Ulla Näppä, Marie Häggström

**Affiliations:** 1 Department of Health Sciences, Mid Sweden University, Östersund, Sweden; 2 Department of Health Sciences, Mid Sweden University, Sundsvall, Sweden

**Keywords:** Bereavement support, Chronic Sorrow, Education, Quantitative and Qualitative methods, Relatives

## Abstract

**Background:**

While experiencing grief following the death of a close relative is a universal experience, the grieving process and the perceived value of bereavement support vary between individuals. While research suggests that most grief reactions are considered normal and diminish over time, some individuals experience prolonged or complicated grief. Knowledge regarding which factors are associated with grief and how bereavement support is perceived by bereaved individuals is limited.

**Aim:**

This study aimed to explore factors associated with grief reactions after the death of a close relative and to describe how bereavement support was perceived by bereaved individuals.

**Methods:**

A quantitative cross-sectional design, including qualitative analysis of written comments was employed. The study’s population was made up of 75 bereaved relatives of patients cared for by a palliative care team based in rural Northern Sweden (response rate 60%). Participants completed the Traumatic Grief Inventory Self-Report Plus (TGI-SR+, Swedish version). Statistical analyses included independent t-tests and analysis of variance (ANOVA). The qualitative component consisted of 51 written comments analysed using inductive manifest content analysis.

**Results:**

Participants whose loss had occurred within the previous 12 months had significantly higher grief scores than those who had been bereaved for up to 2 years earlier. Overall, grief diminished over time, and only one item – longing and yearning for the deceased – reached pathological levels among those bereaved for less than 12 months. The qualitative findings revealed an overarching theme: the art of surviving. Participants described organised bereavement groups, professional listeners, family, friends, and even financial backing as meaningful forms of support. Some reported having no need for formal support, while a few found that talking about the loss increased their distress.

**Conclusion:**

Grief generally decreases over time, and no demographic factors are identified as reducing grief. Bereavement support does not necessarily need to be professional; informal and individual coping strategies may be equally important. Health care professionals should be educated to identify individuals in need of support and to provide person-centred bereavement care.

**Supplementary Information:**

The online version contains supplementary material available at 10.1186/s12904-026-02140-x.

## Introduction

Death is inevitable, and when it comes, it will leave relatives behind, most of whom experience grief. Grief has been widely studied and described as intense emotional reactions to loss of a beloved person to death [1, 2]. However, grief is a universal phenomenon, connected to many kinds of losses, not only to death. As described by Guldin and Leget (2024) in the Integrated Process Model of loss and grief, grief is also related to other types of loss and experiences, such as the loss of a relative to dementia or the loss of relationships, roles, functions or ideals [3]. Research on grief mostly falls within the psychological field and often defines the grieving process as pathologic [4, 5]. Consequently, various therapies have been developed to promote healing from grief; however, most grieving individuals do not suffer from complicated grief [6]. Instead, grief may lead to personal growth and a deeper understanding of life. Hence, grief is a deeply personal experience rooted in the loss of a specific person, identity, meaning or other core value in life. It should be understood through five dimensions: physical, emotional, cognitive, social and, spiritual [3]. 

Sorrow is a feeling connected to loss and grief. Loss may result from different events, such as being the parent of a disabled child or being a relative to a person with severe, chronic illness, which entails an unending list of caregiving responsibilities. In such situations, sorrow has no predictable end and can become chronic. Loss can also be more circumscribed, such as the death of a loved one [7]. Therefore, grief is the experience that follows loss such as the death of a loved one. It normally involves an urge to look back, to cry and, search for what is lost as well as a conflicting urge to look forward and to see what may emerge – a way of discovering what can be carried forward from the past [8]. Normal sorrow and grief have an end [7, 8] most people will experience sorrow and grief after the death of a loved one, whether the death was imminent or occurred slowly. But sorrow and grief affect people in different ways. Some individuals handle it with calmness and take it as it comes, while others cannot stop thinking about their deceased loved one. In such cases, grief may become severe and prolonged [9–11] potentially developing into a chronic, pervasive sorrow [7]. According to the Middle-Range Theory of Chronic Sorrow, chronic sorrow is distinguished from timebound grief. Individuals experiencing chronic sorrow periodically re-experience pervasive grief-related feelings similar to those they felt when first confronted with the loss. This sorrow becomes cyclical, and situations in which individuals encounter the ongoing disparity can trigger new grief-related responses. Due to this cyclical nature, periods of satisfaction and happiness coexist with the episodes of renewed grief, thereby preventing the sorrow from becoming incapacitating [7]. The needs for bereavement support typically follows sorrow and grief as a way of coping [3]. Increased awareness of grief which is no longer a taboo subject, has allowed bereavement to more openly take place in society. This has led to the provision of bereavement support within the context of societal openness [9, 11–15]. Having the opportunity to tell the story of the illness and death of the deceased person has been identified as a factor that relives and alleviates grief [10, 12]. This is described in the theory as the process of contingency. An experience of contingency is caused by a life event that conflicts with a person’s view of world and life goals. It raises existential crises and questions. These experiences may urge the person to reinterpret their own life story, including the disruptive event [16]. Disease severity and behavioural changes among carers of patients with incurable diseases have previously been identified as risks for anticipatory grief [17]. Continuity of care for the dying person at the end of their life is an important aspect of bereavement support. For example, action plans for care and proactive care planning drawn up by healthcare professionals, have been described as preferable for relatives to home-dwelling patients at the end of life [18]. In addition, bereavement support provided in preparation for the imminent death of a relative can reduce the adverse consequences of the death, such as anxiety and stress [19, 20]. In Sweden, palliative care should be provided to all persons with palliative care needs, as well as their relatives and family. When a person dies, the relatives and family members hold a special position. At least one bereavement conversation should be offered to promote quality of life. The health status of the bereaved individuals is considered to be of moderate severity, and such conversations are therefore very important, according to National Board of Health and Welfare’s recommendation group [21]. Bereavement support can be provided individually or in groups, in the form of conversations, bereavement groups, or guidance, by hospitals, primary health care services, social service, the church, or voluntary organisations depending on personal needs and local resources [22]. Despite the availability of various forms of bereavement support for relatives and family caregivers after death, knowledge about possible grief reactions, and how such support is perceived remain limited.

### Aim

This study aimed to explore factors associated with grief reactions after the death of a close relative and to describe how bereavement support was perceived by bereaved individuals.

## Methods

The study is part of a larger project comprising four studies that has the overall aim of describing bereaved individuals’ experiences of grief and support following the death of a close relative. This study employed a quantitative cross-sectional design to identify factors associated with grief reactions after the death of a close relative. Open-ended questions were analysed using qualitative content analysis [23, 24] to explore how the bereavement support offered was perceived. This approach enabled the collection and analysis of data from both designs, the integration of findings and the drawing of inferences relevant to nursing research [25]. 

### Participants and settings

Participants in the study were relatives to persons cared for by a palliative care team at a hospital in rural Northern Sweden. This team cares for approximately 150 persons with palliative care needs annually, almost all of whom have relatives. All the deceased’s closest relatives, regardless of their relationship, were invited to participate in this study between February 13, 2025, and September 5, 2025. All potential participants had previously received an invitation to take part in bereavement groups held at the hospital. These bereavement groups consisted of five scheduled meetings with predetermined programmes in which 5–10 participants met to share their grief with others in similar situations. The groups were led by a deacon from the hospital church and a social worker from the hospital, both of whom had many years of experience supporting individuals after loss. For reasons unknown, not all individuals invited to the study chose to participate in the bereavement groups.

**Fig. 1:**
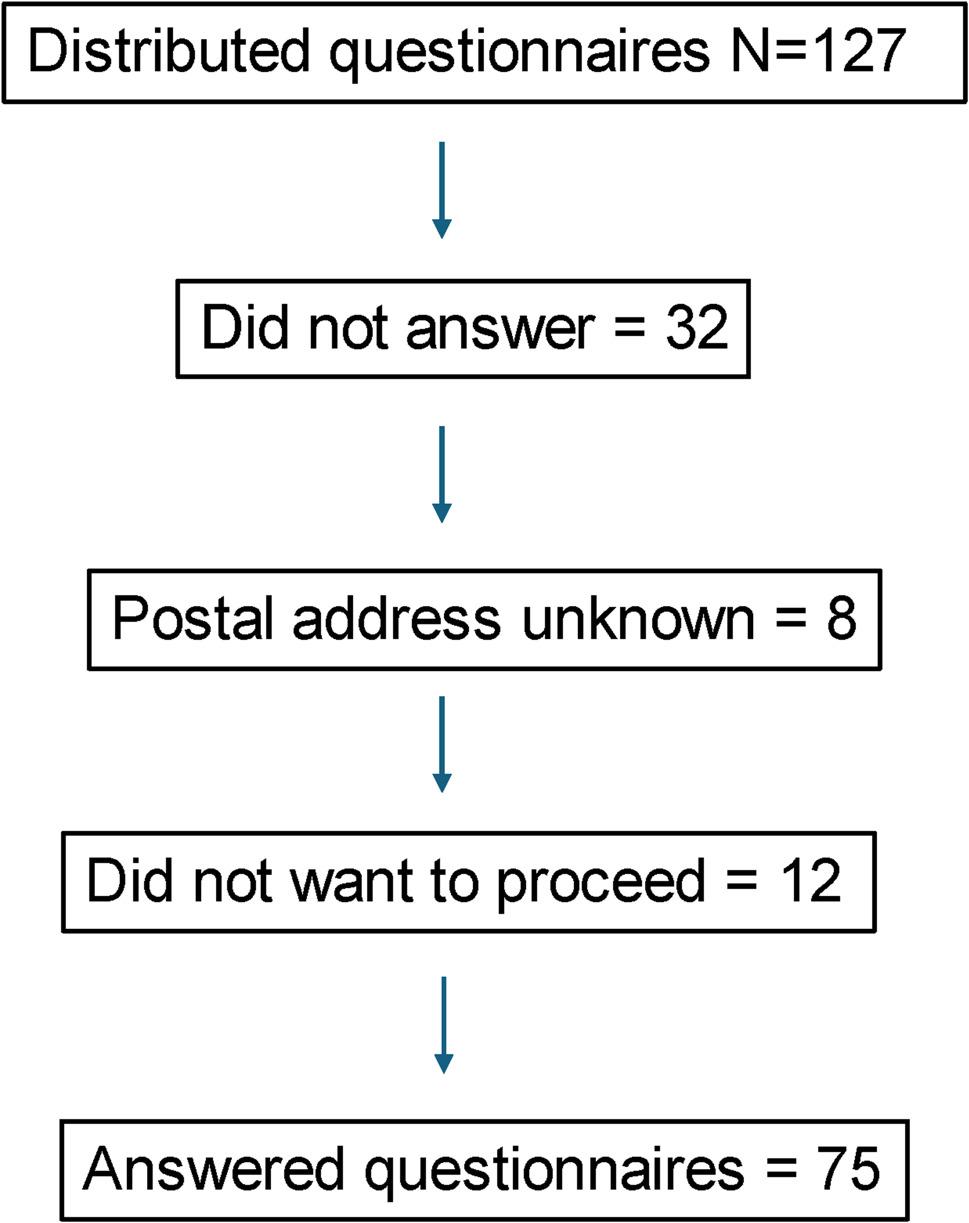
Flowchart of participant recruitment and response

The surgery clinic, with the coordination of the deacons and social workers, granted permission to share the relatives’ contact information. Invitations to participate in the study were sent by the first author. These invitations included an information letter about the study, an informed consent form, a paper-based questionnaire and a pre-stamped return envelope. These materials were sent to all 127 individuals who had been invited to one of four bereavement groups during a two-year period. No additional contact was made by the researchers beforehand the invitation. After two weeks, a reminder letter was sent to individuals who had not responded initially. Third reminders were not sent in consideration of the vulnerability associated with bereavement. Recipients were allowed to complete the questionnaire, return the informed consent form to decline participation or not respond at all (see Fig. 1).

### Questionnaire

Participants completed the Traumatic Grief Inventory Plus (TGI-SR+) [26], (Supplementary Material) Swedish version TGI-SR+(Swedish) (Supplementary Material) which measures the intense and prolonged grief reactions that severely impact daily functioning. The questionnaire was developed to capture potential markers of disturbed grief according to diagnostic criteria for Persistent Complex Bereaved Disorder (PCBD) in the DSM-5, and Prolonged Grief Disorder (PGD) in the DMS-5-TR and ICD-11. In development of the TGI-SR+ “natural cause of death, e.g. illness”, was one of five investigated reasons for traumatic grief (*n* = 208/548 persons) [26]. These diagnostic frameworks strongly influence research and clinical decision-making related to the management of grief [27]. The TGI-SR was developed as a self-report instrument to assess symptoms derived from the diagnostic criteria outlined above. The items in the questionnaire measure symptoms grouped into different criteria domains (see Table 1). The instrument has been shown to be robust, reliable, valid and, applicable for assessing disturbed grief across different contexts [26]. 

**Table 1:** Items in TGI-SR+(Swedish) differentiated to criteria measuring PCBD and PGD

Symptoms measured for PCBD with DSM-5
Criterion B – separation distress (items 1, 2, 3 and 14)
Criterion C – reactive distress, social identity disruption (items 4–11 and 15–18)
Criterion D – functional impairment (item 13)
Symptoms measured for PGD with DSM-5
Criterion B – separation distress (item 1 and 3)
Criterion C – cognitive, emotional and behavioural (items 6, 9, 10, 11, 18, 19 and 21) Criterion C4 – intense emotional pain (e.g., anger, bitterness, sorrow) related to the death of a loved one (items 2 and 8)
Criterion D – functional impairment (item 13)
Symptoms measured for PGD with ICD-11
Criterion B – separation suffering (items 1 and 3)
Criterion C – cognitive, emotional and behavioural (items 2, 5, 8, 9, 10, 16, 19, 20, 21 and 22)
Criterion D – functional impairment (item 13)

The TGI-SR + and TGI-SR+(Swedish) consists of 22 items assessing different grief reactions, with responses rated on a Likert scale ranging from 1 to 5: *never – rarely – sometimes – frequently – always*. The maximum possible total score is 110. A symptom was considered endorsed when rated “frequently” or “always” (scores 4 or 5). Responses of *frequently* and *always* were therefore counted as indicators of prolonged grief reactions. According to the instrument’s developers, the cut-off score for identifying possible cases of disturbed grief, involves the sum of all 22 TGI-SR+ items is being greater than or equal to 71 [26]. In addition to the TGI-SR+, a set of study-specific questions was included to collect background information. These items covered gender, age, employment status, educational level (categorized into predefined levels); time since loss, assessed using a free-text response, in which participants were asked to report in their preferred unit (e.g., days, weeks, months, or year); relationship to the deceased (e.g., partner, parent, child); and, whether any form of bereavement support had been provided (assessed with dichotomous item, yes/no, with a follow-up question on the type of support provided if the answer was yes). The following open-ended question was also added: *If you received bereavement support*,* please describe how it helped you through your bereavement*.

### Quantitative analysis

The questions were initially grouped for analysis according to participants’ PCBD and PGD scores. This allowed for the determination of which grief criteria domains scored highest. To examine whether participants’ background characteristics were associated with differences in high scores, the analysis included independent t-tests or ANOVA, depending on the variables analysed. A *p*-value of *≤* 0.05 was considered statistically significant. The dropout rate in the study was 41%; of the 127 distributed questionnaires, 75 were answered (see Fig. 1).

To further examine whether time since loss influenced grief levels, or whether some individuals were at risk of chronic sorrow, the data were dichotomised into time since loss of one year or less versus time since loss of more than a year.

### Qualitative analysis

A content analysis with an inductive design following that developed by Graneheim and Lundman [23, 24] was conducted on the 51 written comments responding to the question *If you received bereavement support*,* please describe how it helped you through your bereavement*. Content analysis is suitable for this type of data, as the written comments were already condensed and, to some extent, abstracted by each participant. At this level, a manifest analysis described what the text is about, allowing the visible and explicit meanings of all participants’ comments to be identified. As the comments were written responses and because no further contact was made with the participants, it was not possible to explore the comments’ underlying latent meanings [28]. The comments were read and reread by the researchers to facilitate familiarization with the content. The comments were then divided into meaning units – some of them as a whole and others split into smaller units according to their content. The meaning units were condensed into shorter formulations that remained close to the original text. Thereafter, the condensed meaning units were labelled with codes abstracted from the whole meaning units. These codes were compared based on similarities and differences and sorted into subcategories and categories constituting the manifest content, in line with this method of content analysis [24]. The development of categories was guided not the number of comments but the information power in the comments [29]. This analytic process moved back and forth between the data and the emerging structure to ensure alignment with the study’s aim and to enhance credibility. The excerpts in the Results section are identified by item number, time since loss and relationship to the deceased.

## Rigour

The first author has extensive experience working in palliative care, while the second has substantial background in intensive care. In addition, the authors carry complementary expertise in research design: the first author is more familiar with quantitative methods, while the second is more comfortable with qualitative methods. This combination was advantageous for this project. Both authors are accustomed to meeting people experiencing sorrow, albeit from different care contexts. The authors’ preunderstandings were discussed and reflected upon; as both care settings involve close interactions with relatives, these discussions were considered fruitful. The data, analytic processes and, emerging results were triangulated during both the quantitative and qualitative analyses through the researchers’ differing perspectives. The study’s findings were continuously judged, juxtaposed and, evaluated to enhance reliability, validity and trustworthiness [25]. 

## Ethics

This study involved individuals who had experienced vulnerable life events. For this reason, invitations to participate in the study were sent no more than twice to each patient. The invitation letter informed participants that they could withdraw their consent to participate into the study at any time without providing a reason. The informed consent form clearly stated the first author’s contact information. Participants who experienced distress related to the study were invited to contact the researcher; if necessary, information about professional bereavement support was provided in accordance with the Declaration of Helsinki [30]. None of the researchers had any prior relationships with the participants. Moreover, the study was approved by the Swedish Ethical Review Authority, Regional Ethics Committee in Umeå, Department of Other Research, (approval number 2018–323 − 31).

## Results

Quantitative analysis.

The quantitative analysis aimed to explore factors associated with grief reactions after the death of a close relative. Of the 127 individuals invited to participate in the study, 75 responded the TGI-SR+(Swedish) questionnaire (Fig. 1). Of these 75 participants, 19 (25.3%) had experienced the loss of a loved one a year or less from their participation in the study (time since loss ranging from 7 to 12 months), while 56 participants (74.7%) had experienced the loss more than one year prior to the study (13–48 months). For demographics, see Table 2.

**Table 2:** Demographic of participants, *N* = 75

Time since loss in months	*N* = 75 (%)
	7–48 months
Gender	
Male Female Declined to state Missing	28 (38.7) 45 (60.8) 1 (1.4) 1 (1.4)
Relation to deceased	
Partner Child Parent Other	46 (48.4) 21 (28.0) 5 (6.7) 3 (4.0)
Age in years	32–88 years Mean 66.15
Age groups in years	
31–50 51–70 71 –	11 (14.7) 29 (38.7) 35 (46.7)
Working status	
Working Unemployed Retired Missing	29 (30.5) 1 (1.4) 44 (59.5) 1 (1.4)
Education	
Primary school Elementary School Upper secondary school Vocational education University education missing	6 (8.5) 2 (2.8) 22 (31.0) 9 (12.7) 32 (45.1) 4 (5.6)
Bereavement support	
Yes, I got some kind of support No, I did not get support Missing	45 (60.8) 29 (39.2) 1 (1.4*)
Total TGI-SR+(Swedish) score	19–90 Mean 46.13

When dichotomised by time since loss (experiencing loss within the past year versus experiencing loss more than a year ago), the two groups were largely homogenous with respect to demographic variables. However, a statistically significant difference was found in the total of TGI-SR+ score among participants who had experienced the loss within the past year (see Table 3).

The results were compared according to the symptom criteria for PCBD and PGD to determine which symptoms were associated with different demographic groupings. PCBD was measured for three criteria in the questionnaire. For Criterion B, *separation distress*, the only difference was related to time since loss, with higher scores found among participants who had experienced loss within past year. For Criterion C, *reactive distress/social identity disruption*, no significant differences were found. For Criterion D, *functional impairment*, scores decreased over time, however, no differences were deemed statistically significant. Bold p-values indicate statistical significance. (Table 3).

**Table 3:** Demographic of participants dichotomised by time since loss, *N* = 75

	Loss during the last year *N* = 19 (%)	Loss more than a year ago *N* = 56 (%)	*p*-value
Time since loss in months	7–12 months	13–48 months	**< 0.001**
Gender			
Male Female Declined to state Missing	8 (42.1) 11 (57.9)	20 (35.7) 34 (60.7) 1 (1.8) 1 (1.8)	0.867
Relation to deceased			
Partner Child Parent Other	14 (73.7) 4 (21.1) 1 (5.3)	32 (57.1) 17 (30.4) 4 (7.1) 3 (5.4)	0.773
Age in years	43–88 years Mean 66.11	32–88 years Mean 66.16	0.988
Age groups in years			
31–50 51–70 71 –	2 (10.5) 9 (47.4) 8 (42.1)	9 (16.1) 20 (35.7) 27 (48.2)	0.733
Working status			
Working Unemployed Retired Missing	6 (31.6) 13 (68.4)	23 (41.1) 1 (1.8) 31 (55.4) 1 (1.8)	0.613
Education			
Primary school Elementary School Upper secondary school Vocational education University education missing	2 (10.5) 3 (15.8) 3 (15.8) 9 (47.4) 2 (10.5)	4 (7.1) 2 (3.6) 19 (33.9) 6 (10.7) 23 (41.1) 2 (3.6)	0.507
Bereavement support			
Yes, I got some kind of support No, I did not get support Missing	11 (57.9) 7 (36.8) 1 (5.3)	34 (60.7 22 (39.3)	0.976
Total TGI-SR+(Swedish) score	32–90 Mean 53.37	19–85 Mean 43.7	**0.022**

PGD was measured using five criteria in the questionnaire. No means values did reach pathologic levels for any criterion. The analysis showed that Criterion B in both DSM-5-TR and ICD-11: *separation distress* or *separation suffering* decreased significantly over time. Similarly, Criteria C in both DSM-5-TR and ICD-11: *cognitive*,* emotional and behavioural*, and Criterion C4 in DSM-5-TR; *intense and emotional pain related to death* decreased over time. Criterion D, *functional impairment*, decreased during time, although the differences were not statistically significant. The only significant difference between men and women was found for Criterion C: *cognitive*,* emotional and*,* behavioural* for ICD-11. The only significant relationship to the deceased was observed for Criterion C4, *anger*,* bitterness*,* sorrow*. In terms of age groups, significant differences were found for Criterion C4 and Criterion C according to ICD-11. Overall, the demographic factor most strongly associated with grief symptom criteria was time since loss. Bold values indicate statistical significance (see Table 4). Assumptions for parametric analyses were examined prior to conducting the independent t-tests and ANOVAs. Homogeneity of variance was assessed using Levine’s test and was met for all analyses. In addition, the data were inspected for approximate normality.

**Table 4:** TGI-SR+ (Swedish) results compared with demographic variables (*p*≤.05)

	Time since loss of one year or less versus time since loss of more than a year^1^ *p*-value	Gender (dichotomised to male or female)^1^ *p*-value	Relationships^2^ *p*-value	Age Groups^2^ *p*-value	Working status^2^ *p*-value	Education^2^ *p*-value	Received bereavement support ^1^ *p*-value
Persistent Complex Bereaved Disorder (PCBD)							
Criterion B (i.e. separation distress)	**0.015**	0.357	0.165	0.337	0.338	0.159	0.102
Criterion C (i.e. reactive distress/social identity disruption)	0.074	0.381	0.057	0.053	0.213	0.261	0.122
Criterion D for all dimensions (i.e. functional impairment)	0.185	0.496	0.666	0.134	0.581	0.250	0.748
Prolonged Grief Disorder (PGD)							
Criterion B with DSM-5 and ICD-11 (i.e. separation distress)	**0.002**	0.656	0.126	0.488	0.162	**0.031**	0.081
Criterion C with DSM-5 (i.e. cognitive, emotional and behavioural)	**0.028**	0.546	0.252	0.114	0.197	0.152	0.295
Criterion C4 with DSM-5 (i.e. anger, bitterness and sorrow)	**0.031**	0.539	**0.049**	0.052	0.911	0.517	**0.035**
Criterion C with ICD-11 (i.e. cognitive, emotional, and behavioural)	**0.015**	**0.046**	0.097	.**023**	0.482	0.341	0.101
Criterion D for all dimensions (i.e. functional impairment)	0.185	0.496	0.666	0.134	0.581	0.250	0.748

^1^ Independent t-test, ^2^ANOVA

The overall results from the quantitative analysis showed that distress scores differed according to time since loss. Participants who had experienced the loss within the past 12 months prior to the study reported higher levels of distress than those whose loss had occurred more than 12 months earlier. However, only one item – item no. 3, *I found myself longing or yearning for the person who had died* – reached the threshold for symptom endorsement among bereaved participants based on scores on the Likert scale, where a symptom was considered endorsed when rated “frequently” or “always” (score of 4 or 5). Item-level comparisons were conducted using independent t-tests to identify which specific items contributed to distress within each criterion; these results are presented in Table 5. Bold *p*-values indicate statistical significance. It should be noted that item 2 is classified under Criterion B according to PCBD in Criterion B and according to PGD in Criterion C.

**Table 5:** Significant differences (*p*<.05) by time since loss ≤ 1 year vs. > 1 year

Criterion and items	Item	*p*-value*	Mean score of items when loss *≤* 12 months	Mean score of items when loss > 12 months
Criterion B – separation distress (items 1, 2, 3 and 14)	2. I experienced intense emotional pain, sadness or, pangs of grief 3. I found myself longing or yearning for the person who died	**0.032** **< 0.001**	3.0 4.37**	2.46 3.36
Criterion C – reactive distress, social identity disruption (items 4–11 and 15–18) cognitive, emotional and behavioural (items 6, 9, 10, 11, 18, 19 and 21) cognitive, emotional and behavioural (items 2, 5, 8, 9, 10, 16, 19, 20, 21 and 22)	5. I had trouble accepting the loss 11. I felt that life is unfulfilling or meaningless without him/her 21. It felt as if a part of me has died along with the deceased	.**012** .**022** .**004**	3.37 2.84 3.05	2.48 2.16 2.09
Criterion C4 – intense emotional pain (e.g., anger, bitterness and sorrow) related to the death (items 2 and 8)	2. I experienced intense emotional pain, sadness, or pangs of grief	.**032**	3.0	2.46

*Independent T-test **Score *≥* 4 = scoring 4 or 5, frequently or always, respectively, on the Likert scale

### Qualitative analysis

The qualitative analysis aimed to describe how the bereavement support offered was perceived. A content analysis was conducted based on a total number of 51 written comments from the questionnaires. A total of 45 participants reported that they had received some form of bereavement support. Participants described support as supportive conversations, provided by healthcare professionals, social workers or church, participation in bereavement groups arranged by hospital or church, speaking with relatives, trauma-focused cognitive behavioural therapy, and survivor’s pension.

**Fig. 2:**
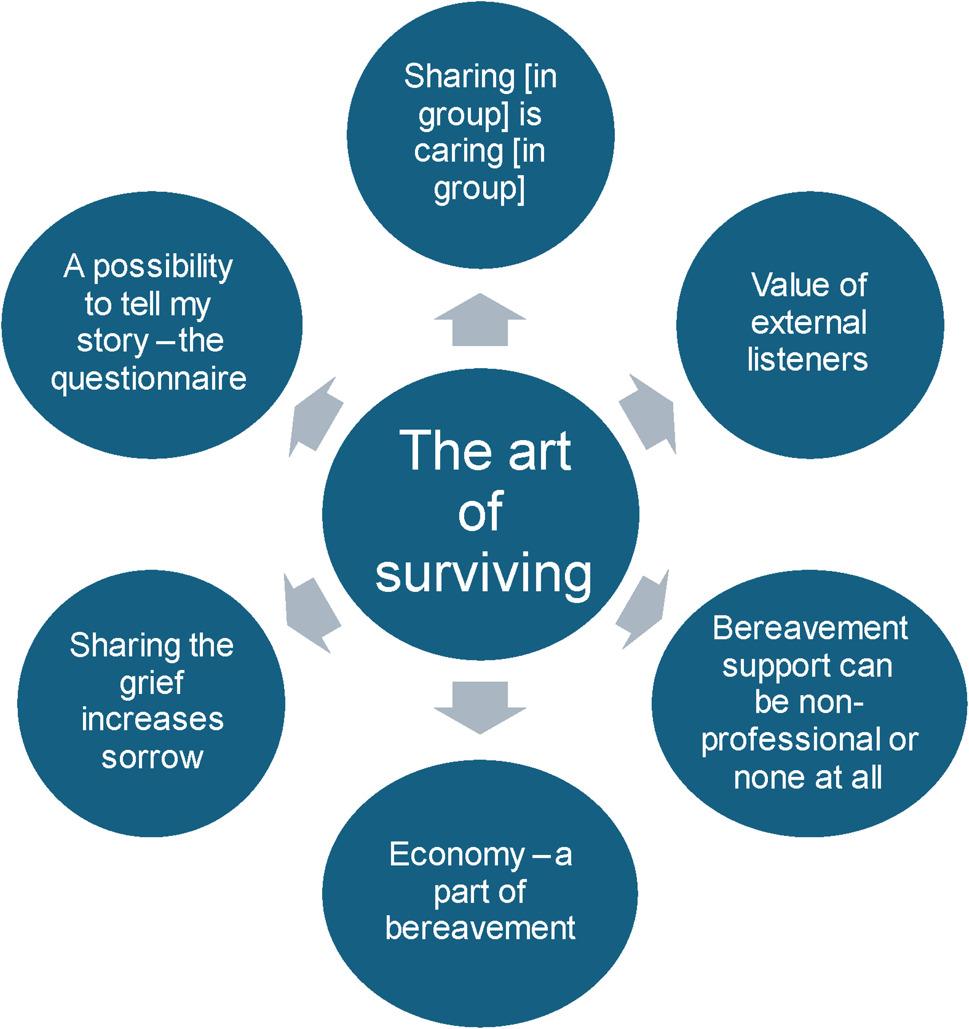
The overarching theme “The art of surviving” and its related categories

The manifest analysis revealed an overarching theme *– The art of surviving –* derived from six categories (Fig. 2.)

The overarching qualitative theme indicated that almost all bereaved participants who responded the question *If you received bereavement support*,* please describe how it helped you through your bereavement* described strategies for coping with the future. Participants expressed a desire to continue with life and to survive their period of sorrow. Their experiences ranged from benefitted from organised bereavement support to managing their grief independently or claiming that they had no need for bereavement support.

### Sharing [in group] is caring [in group]

Participants reported that they had been invited to bereavement groups. These invitations were appreciated, as the groups had established organisations with clear routines. The meetings of these groups addressed participants’ emotions, and the participants’ bereavement processes progressed through the introduction of new themes at each session. In a few cases, the leaders’ competence at professional conversational support was perceived as insufficient. Nevertheless, the participants generally appreciated the meetings and found them to be valuable. Participants also found the mutual discussions supportive and perceived the group leaders as experienced. Listening to others allowed them to distance themselves from their own sorrow while simultaneously fostering compassion and understanding. Participants felt that they did not have to explain everything that they were feeling. This created a sense of not being alone and an understanding that life must go on. One participant described their experience as follows:

“Yes, we had a bereavement group for processing grief where we got to hear about how others had experienced it and how they coped with the situation. It felt comforting to know that I am not alone and that there are others who have ended up in similar situations. We were able to talk, encourage one another, and try to focus on the idea that life must go on.” (P 33, 24 months, wife).

Some people also formed new friendships by participating in these groups. These friendships were described as among the most meaningful outcomes of the bereavement groups. One participant wrote, “We are still five persons who see each other every month” (P18, 36 months, wife), even though it had been a year and a half since her loss.

### Value of external listeners

Some participants who had not participated in the bereavement groups still expressed a need for professional listeners. Most of them had met with a professional bereavement counsellor or health care counsellor, either once or on several occasions. Others reported receiving support from the local church. These participants found it easier to put words to their thoughts and feelings when alone with a counsellor. One participant pointed out that beginning the bereavement process before the loved one’s death was beneficial for coping the upcoming sorrow.

“I had support throughout my mother’s illness and, death as well as the period that followed. I had individual conversations with a counsellor, a priest, and a behavioural scientist. I also participated in the hospital’s bereavement group and a 12-step group through the church” (P27, 24 and 36 months, daughter of two deceased parents).

In this case, the participant had lost both her parents and had taken part in both individual counselling and group-based support.

External listeners also were described as a means of obtaining relief from strained relationships or difficult behaviour among relatives. In addition, a few participants who needed support found that although they could have benefitted from having someone to talk to, they did not receive such support.

### Bereavement support can be non-professional or none at all

Participants found that bereavement support did not necessarily need not to be professional. Many participants reported receiving substantial support from relatives and friends. Some lived too far from the hospital for participation in bereavement groups to be feasible. Another source of support mentioned was the formation of a new intimate relationship. One person, aged 82, wrote “New love has helped me a lot in my grief” (P51, 14 months, male).

Other participants stated that they had been offered bereavement support but chose to decline these invitations. Most of these participants expressed no perceived need for support, while others described the timing of such invitations to be inappropriate.

### Economy – a part of bereavement

Some of the participants identified financial support funding, such as survivor’s pension, as a form of bereavement support. This type of support did not provide emotional relief but made it possible for these individuals to remain in their homes. One participant noted: “Monthly pension is no help in the bereavement process – it is more like a reminder of the loss every month”. (P2, 33 months, son).

### Sharing the grief increases sorrow

Several participants reported that bereavement counselling worsened their situations. Experiences related to the relative’s death and feelings of guilt became deeply fixed in their memory. Attempts to participate in the bereavement groups trigged reminders of the dying process and the moment of death. One participant wrote, “The guilt I felt about thinking of myself instead of staying with him on the last evening only worsened after the bereavement support. My guilt caused me to attempt suicide, which was followed by my involuntary admission [to a psychiatric clinic]” (P39, 18 months, daughter).

### A possibility to tell my story – the questionnaire

Participation in the study itself was described as a way easing sorrow. Some participants wrote several sentences about their situation, claiming that the questionnaire functioned as a kind of counsellor. Several patients wrote about the progression of the deceased loved one’s illness, others about their feelings of sorrow after the loss, while others still perceived shortcomings in the healthcare system. An 88-year-old participant wrote:

“It feels difficult to answer. I remember and experience a sense of melancholy, ‘like my youth,’ for example ‘our small children’. Life goes on. It changes. My mother and grandmother went through it and showed that you can manage. I know that I can be happy and grateful. Two years have passed. Osteoarthritis and dizziness, among other things, make it difficult, of course. But I receive help and experience joy. It is so different. Dementia and Alzheimer’s, for example. My husband died ‘while still alive’; he was able to stay at home for a long time and appreciated that. It was good for me as well.” (P37, 24 months, wife).

## Discussion

This study aimed to identify the factors that could reduce grief after the death of a close relative and to describe how the bereavement support offered to these individuals was perceived by them. The quantitative results showed that the only difference between participants was related to time since loss. Levels of sorrow and grief were higher closer to the time of loss and decreased over time. Only the item *I found myself longing or yearning for the person who had died* reached pathological levels among participants who had experienced the loss within the past 12 months. These findings confirm earlier research showing that sorrow is generally a normal response to loss and tends to diminish over time [3]. The qualitative results demonstrated that most participants were positive about receiving bereavement support to help manage their sorrow and grief, although this support did not necessarily need to be professional. According to quantitative results, no participants appeared to be at risk of chronic sorrow, as described in The Middle-Range Theory of Chronic Sorrow [7] which was reflected in constantly low scores on the questionnaire items. However, a few participants reported in the open-ended comments that conversations about their grief worsened their situations. A review by Kazoleas (2025) investigating young widowers’ experiences of loss revealed that bereavement involved more than losing a spouse; hopes and dreams also had to be relinquished, and now choices had to be made in order to move forward. For some individuals, grief shaped new and positive phases of life that were characterised by personal growth, independence and empowerment [31]. This review strengthens the findings in this study, suggesting that although while grief can be long-lasting, it changes and evolves over time. Moreover, this study’s participants cover a wide age range, although most were older than 70 years. No differences were found between age groups in terms of levels of sorrow, indicating that sorrow and grief is not primarily a function of age. However, healthcare professionals encounter these individuals in many situations within society. This may be related to their own illness, when taking a medical history, or during contact to seek answers about the deceased loved one’s illness. Relatives may have a need to tell their stories again [10]. Therefore, the findings are important for healthcare professionals caring of individuals with a limited life expectancy, as the death of a loved one may lead to social consequences, such as disrupted life plans or financial strain for relatives. The Middle-Range Theory of Chronic Sorrow describes chronic sorrow as an experience that returns cyclically so long as the disparity created by the loss remains [7]. If relatives are prepared for grief and bereavement and the possibility of such recurring experiences before the impending death, the risk of chronic sorrow may be reduced. However, individuals differ in how they experience and manage grief. Therefore, maintaining a focus on individual needs is an essential aspect of care for bereaved relatives. An individualised approach to sorrow, a normal process, may help prevent it from becoming chronic. One way to support such an approach is to view grief and sorrow through the lenses of the cognitive, social, spiritual, physical and emotional processes presented by Guldin and Leget (2024) [3] in their work on end-of-life care. This perspective facilitates an understanding of both current and future needs. It enables healthcare professionals to understand how these dimensions interact and to plan care accordingly in encounters with bereaved individuals. Clarifying the grieving process for healthcare professionals may help them navigate and integrate these dimensions in conversations about grief [3]. To achieve this goal, nurses must be prepared and willing to discuss death, dying and bereavement with patients and relatives, both before and after death.

Preventive strategies should be carried out at an even earlier stage to further investigate factors influencing grief reactions, to reduce the risk of chronic sorrow among relatives. Nurses’ attitudes toward end-of-life care have been shown to influence outcomes for both patients and relatives. A systematic review by Alshammari et al. (2022) of registered nurses working in non-specialist palliative care settings has revealed significant findings on symptom management and patient care: however, knowledge of spiritual needs and psychosocial needs was limited, which contributed to nurses’ reluctance to discuss death and dying [32]. Stenman et al. (2023) and Stenman et al. (2024) demonstrated that when nurses assume responsibility for engaging with patients at the end of their lives, the quality of care and nursing improves. Knowledge of spiritual needs fosters trust between patients and nurses and enables confidential conversations [33, 34]. Furthermore, this trust is strengthened when relatives are included in care discussions [35]. Healthcare professionals in palliative care must exert effort at all educational levels to support the development of effective interdisciplinary teams and improve end-of-life care. However, research indicates that undergraduate nursing students often receive inadequate training in death and end-of-life care. If education in conversations with severely ill patients and their relatives is introduced early and reinforced throughout nursing programmes, students may be better prepared to support bereaved individuals in their professional roles. Additionally, digital education and simulation-based training focused on death and bereavement may enhance nursing students’ emotional and psychological preparedness [36, 37]. In Singapore, undergraduate nurses and medical students were jointly invited to participate in discussions in death cafés. The students reported that these cafés provided valuable opportunities for open discussions about coping with encounters with death of patients and for developing communication skills in such situations. Through this experience, the undergraduate nurses and medical students gained greater knowledge of managing palliative and end-of-life care [38]. The present study supports the argument that interdisciplinary education should be incorporated into nursing curricula to prepare students for their future professional practice.

Strengths and limits.

The participants were not representative of the general population, as the sample consisted predominantly of women and individuals with a higher education level. This limits the generalisability of the study’s findings. In addition, the quantitative sample size was relatively small (*n* = 75), which may have reduced the study’s statistical power to detect differences between groups. Given the relatively small sample size, checking assumptions was considered particularly important, and no substantial violations were detected. A strength of the study lies in the combined analysis of quantitative and qualitative data derived from the same data collection, enhanced by the researchers’ proficiency in their respective methodological approaches. The integration of findings from both designs contributed to the study’s interpretive and theoretical consistency, agreement and complimentarity. As this study has not a mixed methods design as the results are not emerged to develop integrated results as described by Flanagan and Beck [39]. However, this approach with combining quantitative and qualitative data enhanced the validity and depth of the results. The quantitative findings, which show that grief diminished over time, are consistent with earlier results [11]. Another strength of this work is the inclusion of written comments concerning bereavement support. Unlike many previous studies, this study did not only examine whether bereavement support was provided, but also how it was perceived and whether it was experienced as meaningful. More than half of the participants provided written comments, including reflections on both professional and non-professional forms of support. This contributed to a broader and more nuanced understanding of grief and sorrow following loss. However, the open-ended question was phrased as follows: “If you received bereavement support, please describe how it helped you through your bereavement.” This wording may imply that the support was expected to be helpful and therefore have influenced the responses. The research team’s complementary clinical experience further strengthened the analysis. The first author’s extensive experience working with bereaved individuals contributed familiarity with the phenomenon under study, while the second author’s background in intensive care offered additional perspective on loss in acute situations. Reflexive discussions during the analytic process revealed that these differing perspectives contributed to a deeper and nuanced interpretation of the findings.

Conducting research involving seriously ill individuals and their relatives may be perceived as controversial. The response rate in this study was 60% which suggests that, for many participants, the perceived benefits of responding outweighed the potential burdens. These participants likely understood the seriousness of the subject and were motivated by ethical principles of beneficence. They may view participation as an altruistic act or an opportunity to contribute to improvements in end-of-life care and thereby help others. Participation in this study may also have benefitted participants in that they received attention. Some studies indicate that individuals’ emotional distress or physical burden may diminish through participation in such research [40]. Participants who responded to this questionnaire took the opportunity to tell their stories about life before the death of their loved one. It is unknown whether these individuals had denied the illness or acknowledged their loved one’s impending death; however, their written comments suggest a need to recount their experiences. Moreover, recalling and narrating the story of the loved one’s dying and death has been shown to be part of the process of working through loss [10, 41, 42]. 

### Ethical considerations

Burke (1999) argued, from the perspective of the theory of chronic sorrow that memories may act as triggers that exacerbate chronic sorrow [43]. Receiving the questionnaire could therefore have trigged increased sorrow among participants possibly experiencing chronic grief. In this study, no information was obtained from individuals who returned the informed consent and marked *“I don’t want to participate”* as their reason for declining participation. Including a follow-up question asking why they declined to participate might have offered the researchers an opportunity to identify individuals who required support from healthcare services.

## Conclusion

This study did not identify specific factors that trigger sorrow or grief after death of a loved one. Instead, it confirmed previous research showing that grief generally diminishes over time. The qualitative findings demonstrated that bereavement support does not necessarily need to be professional. Non-professional support, such as that provided by family and friends, may be equally valuable. Some individuals reported having no need for formal support at all. Others found their own ways of coping; for example, participation in bereavement groups sometimes led to continued contact and self-initiated meetings beyond the structured programme. However, some individuals struggled to manage grief and sorrow following the loss of a loved one. This highlights the importance of educating healthcare professionals about bereavement support and ensuring they have knowledge and competence required to identify and respond individuals in need of this support.

## Supplementary Information

Supplementary Material 1.

Supplementary Material 2.

## Data Availability

The datasets used and/or analysed during the current study are available from the corresponding author on reasonable request.

## Abbreviations

ANOVA: Analysis of variance
DSM: Diagnostic and Statistical Manual of Mental Disorders
ICD: International Classification of Diseases and Related Health Problems
PCBD: Persistent Complex Bereaved Disorder
PGD: Prolonged Grief Disorder
TGI+SR+: the Traumatic Grief Inventory Plus
