# Supplementary material for: Grief after the death of a close relative: a study of associated factors and perceptions of bereavement support
Source: BMC Palliat Care. 2026 May 13;25:138. doi: 10.1186/s12904-026-02140-x (PMC13169666; doi:10.1186/s12904-026-02140-x)
Supplement: Supplementary file 1 — Supplementary Material 1. [file 12904_2026_2140_MOESM1_ESM.pdf]

The Traumatic Grief Inventory-Self Report plus (TGI-SR+)

Manual

Introduction

The Traumatic Grief Inventory-Self Report plus (TGI-SR+) consists of 22 statements about grief reactions, hereafter referred to as items. These items are similar to symptoms of (i) persistent complex bereavement disorder as described in the DSM-5 (APA, 2013), (ii) prolonged grief disorder as described in the revision of the fifth edition of the DSM (DSM-5-TR; APA, 2020), (iii) prolonged grief disorder as included in the ICD-11 (WHO, 2018), and (iv) prolonged grief disorder as proposed by Prigerson et al. (2009). See Table 1 for an overview of the relation between the TGI-SR+ items and these symptoms.

Table 1. Similarities between TGI-CA items and symptoms of disturbed grief.

TGI-CA item	Persistent Complex Bereavement Disorder DSM-5 (APA, 2013)	Prolonged grief disorder DSM-5-TR (APA, 2020)	Prolonged grief disorder ICD-11 (WHO, 2018)	Prolonged grief disorder Prigerson et al. (2009)
1	B3	B2	B2	
2	B2	C4*	C1	
3	B1	B1	B1	B1
4	C11			C1
5	C1		C6	C2
6	C6	C3		C3
7	C8			C4
8	C4	C4*	C3	C5
9	C12	C5	C10	C6
10	C2	C6	C9	C7
11	C10	C7		C8
12				C9
13	D	D	E	E
14	B4			

15	C3			
16	C5		C2	
17	C7			
18	C9	C8		
19		C2	C4	
20			C5	
21		C1	C7	
22			C8	

Note. *These symptoms were assessed with two items. Highest score of one of the two items is used to tap the symptom.

TGI-SR vs. TGI-SR+

This survey was developed in 2019 and based on the 18-item self-report questionnaire the Traumatic Grief Inventory-Self Report (TGI-SR) (Boelen & Smid, 2017; Boelen, Djelantik, de Keijser, Lenferink, & Smid, 2018). The 18 items of the TGI-SR are identical to items 1 through 18 of the TGI-SR+. Four items (item 19 through 22) were added to assess symptoms of prolonged grief disorder according to ICD-11 (WHO, 2018). With the advent of prolonged grief disorder in the DSM-5-TR (2020), TGI-SR items were mapped on these new symptoms.

The participant is asked to rate to what extent each TGI-CA items applies to the participant during the last month on an answer scale with 1 = never, 2 = seldom, 3 = sometime, 4 = often, 5 = always.

Scoring

Summing up the scores on the 22 items results in a total score for disturbed grief. A first validation study indicated that a cut-off score of ≥71 can be used to distinguish disturbed from non-disturbed grief (Lenferink et al., 2022).

Diagnostic scoring rules are helpful to determine whether a person meets criteria for a persistent complex bereavement disorder or prolonged grief disorder. A score of 4 or 5 on an item is indicative of presence of that particular grief symptom.

To meet DSM-5 criteria of persistent complex bereavement disorder at least 1 symptom of the B criterion, at least 6 symptoms of the C criterion, and the symptom of the D criterion needs to be present.

For DSM-5 prolonged grief criteria, the following scoring rule is used: at least one symptom of the B Criterion and at least 3 out of the 8 symptoms of the C Criterion, and the D Criterion symptom should be endorsed.

To fulfill ICD-11 criteria for prolonged grief disorder the following two scoring rules could be applied; the liberal scoring rule, as mentioned by Killikelly and Maercker (2018), that proposes the presence of at least 1 symptom of the B criterion, at least 1 symptom of the C criterion, and the E criterion that suggest presence of prolonged grief disorder or the conservative scoring rule, as stated by Boelen et al. (2019), that advocates that the presence of at least 1 symptom of the B criterion, at least 5 symptoms of the C criterion, and the symptom of the E criterion are indicative of prolonged grief disorder.

To adhere to the criteria for prolonged grief disorder as proposed by Prigerson et al. (2009) the following scoring rule is used: presence of the B criterion, at least 5 symptoms of the C criterion, and the E criterion.

References

American Psychiatric Association (2013). *Diagnostic and statistical manual of mental disorders (5th ed.).* Arlington, VA: American Psychiatric Publishing.

American Psychiatric Association (2020). *Board Approves New Prolonged Grief Disorder for DSM.* Accessed on December 29, 2020 via: https://doi.org/10.1176/appi.pn.2020.11a12

Boelen, P. A., Djelantik, A. A. A. M. J., de Keijser, J., Lenferink, L. I. M., & Smid, G. E. (2018). Further validation of the traumatic grief inventory-self report (TGI-SR): A measure of persistent complex bereavement disorder and prolonged grief disorder. *Death Studies*. doi:10.1080/07481187.2018.1480546

Boelen, P. A., Lenferink, L. I. M., & Smid, G. E. (2019). Further evaluation of the factor structure, prevalence, and concurrent validity of DSM-5 criteria for persistent complex bereavement disorder and ICD-11 criteria for prolonged grief disorder. *Psychiatry Research, 3*, 206-210. doi: 10.1016/j.psychres.2019.01.006

Boelen, P. A., & Smid, G. E. (2017). The traumatic grief inventory self -report version (TGI-SR): Introduction and preliminary psychometric evaluation. Journal of Loss and Trauma, 22, 196–212. doi:10.1080/15325024.2017.1284488

Killikelly, C., & Maercker, A. (2018). Prolonged grief disorder for ICD-11: The primacy of clinical utility and international applicability. *European Journal of Psychotraumatology, 8*(sup6). doi: 10.1080/20008198.2018.1476441

Lenferink, L.I.M., Eisma, M.C., Smid, G.E., de Keijser, J., & Boelen, P.A. (2022). Valid measurement of DSM-5 persistent complex bereavement disorder and DSM-5-TR and ICD-11 prolonged grief disorder: The Traumatic Grief Inventory-Self Report Plus (TGI-SR+). *Comprehensive Psychiatry, 112,* 152281. https://doi.org/10.1016/j.comppsych.2021.152281

Prigerson, H. G., Horowitz, M. J., Jacobs, S. C., Parkes, C. M., Aslan, M., Goodkin, K,. . . Maciejewski, P. K. (2009). Prolonged grief disorder: Psychometric validation of criteria proposed for DSM-V and ICD-11. *PLOS Medicine 6*(8), e1000121.

WHO (2018). *ICD-11. Prolonged Grief Disorder Criteria.* Accessed on October 2, 2019 via: https://icd.who.int/browse11/l-m/en#/http://id.who.int/icd/entity/1183832314

Traumatic Grief Inventory – Self Report Plus (TGI-SR+)

Below several grief reactions are listed. Please indicate how often you have experienced each reaction in the past month in response to the death of your loved one.

		never	rarely	sometimes	frequently	always
1	I had intrusive thoughts or images related to the person who died	☐	☐	☐	☐	☐
2	I experienced intense emotional pain, sadness, or pangs of grief	☐	☐	☐	☐	☐
3	I found myself longing or yearning for the person who died	☐	☐	☐	☐	☐
4	I experienced confusion about my role in life or a diminished sense of self	☐	☐	☐	☐	☐
5	I had trouble accepting the loss	☐	☐	☐	☐	☐
6	I avoided places, objects, or thoughts that reminded me that the person I lost has died	☐	☐	☐	☐	☐
7	It was hard for me to trust others	☐	☐	☐	☐	☐
8	I felt bitterness or anger related to his/her death	☐	☐	☐	☐	☐
9	I felt that that moving on (e.g., making new friends, pursuing new interests) was difficult for me	☐	☐	☐	☐	☐
10	I felt emotionally numb	☐	☐	☐	☐	☐
11	I felt that life is unfulfilling or meaningless without him/her.	☐	☐	☐	☐	☐
12	I felt stunned, shocked, or dazed by his/her death.	☐	☐	☐	☐	☐
13	I noticed significant reduction in social, occupational, or other important areas of functioning (e.g., domestic responsibilities) as a result of his/her death	☐	☐	☐	☐	☐
14	I had intrusive thoughts and images associated with the circumstances of his/her death	☐	☐	☐	☐	☐
15	I experienced difficulty with positive reminiscing about the lost person	☐	☐	☐	☐	☐
16	I had negative thoughts about myself in relation to the loss (e.g., thoughts about self-blame)	☐	☐	☐	☐	☐
17	I had a desire to die in order to be with the deceased	☐	☐	☐	☐	☐
18	I felt alone or detached from other individuals.	☐	☐	☐	☐	☐
19	It felt unreal that he/she is dead	☐	☐	☐	☐	☐
20	I put an intense blame on others because of his/her death	☐	☐	☐	☐	☐
21	It felt as if a part of me has died along with the deceased	☐	☐	☐	☐	☐
22	I had difficulties experiencing positive feelings	☐	☐	☐	☐	☐

Citation TGI-SR+:

Lenferink, L.I.M., Eisma, M.C., Smid, G.E., de Keijser, J., & Boelen, P.A. (2022). Valid measurement of DSM-5 persistent complex bereavement disorder and DSM-5-TR and ICD-11 prolonged grief disorder: The Traumatic Grief Inventory-Self Report Plus (TGI-SR+). *Comprehensive Psychiatry, 112,* 152281. https://doi.org/10.1016/j.comppsych.2021.152281
