# Supplementary material for: Grief after the death of a close relative: a study of associated factors and perceptions of bereavement support
Source: BMC Palliat Care. 2026 May 13;25:138. doi: 10.1186/s12904-026-02140-x (PMC13169666; doi:10.1186/s12904-026-02140-x)
Supplement: Supplementary file 2 — Supplementary Material 2. [file 12904_2026_2140_MOESM2_ESM.docx]

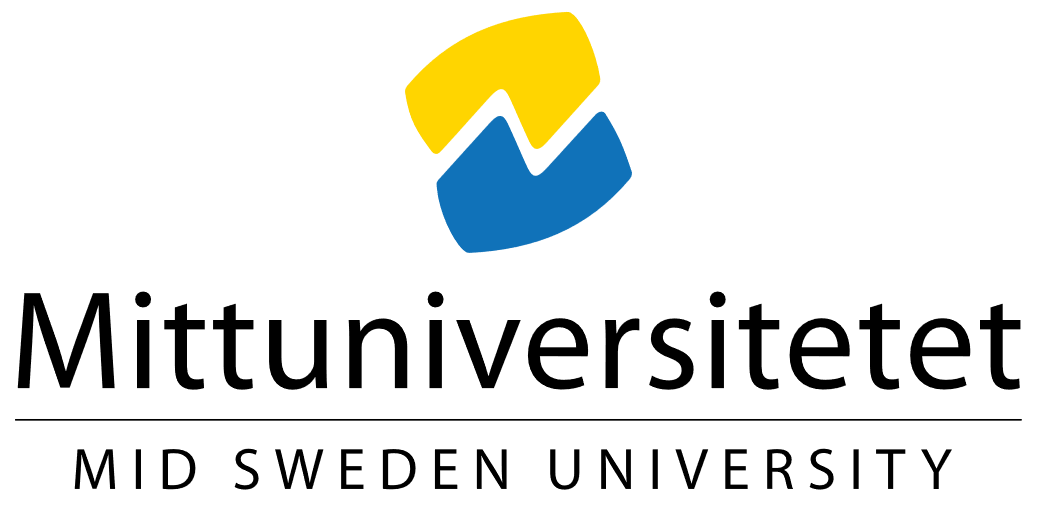
Nedanstående enkät är ett instrument för att bedöma risk för långvarig sorgestörning eller långvarig komplex sorgestörning. I enkäten beskrivs olika sorgereaktioner. Kryssa om du fortfarande upplever någon av följande reaktioner som följd av din närståendes död. Besvara också frågorna om din bakgrund. *Skicka tillbaka enkäten i det portofria svarskuvertet.*

| Traumatisk sorgenkät  svensk översättning av Traumatic Grief Inventory Plus (TGI-SR+) <https://osf.io/vx825>  Sätt kryss i rutan som stämmer med din känsla. | | | | | | |
| --- | --- | --- | --- | --- | --- | --- |
|  | **Påstående** | **Aldrig** | **Sällan** | **Ibland** | **Ofta** | **Alltid** |
| 1 | Jag har påträngande tankar eller bilder relaterade till personen som dog. |  |  |  |  |  |
| 2 | Jag upplever intensiv känslomässig smärta, ledsenhet eller sorg |  |  |  |  |  |
| 3 | Jag känner en längtan eller stark saknad efter den person som dog |  |  |  |  |  |
| 4 | Jag känner förvirring över min roll i livet eller som om jag inte vet vem jag är |  |  |  |  |  |
| 5 | Jag har svårt att acceptera förlusten |  |  |  |  |  |
| 6 | Jag undviker platser, föremål eller tankar som påminner mig om att personen jag förlorat hade dött |  |  |  |  |  |
| 7 | Det är svårt för mig att lita på andra |  |  |  |  |  |
| 8 | Jag känner bitterhet eller ilska över hans/hennes död |  |  |  |  |  |
| 9 | Jag känner att det är svårt för mig att gå vidare (t.ex. att få nya vänner, intressen) |  |  |  |  |  |
| 10 | Jag känner mig känslomässigt avstängd |  |  |  |  |  |
| 11 | Jag känner att livet är tomt eller meningslöst utan honom/henne |  |  |  |  |  |
| 12 | Jag känner mig bedövad, chockad eller omtumlad över hans/hennes död |  |  |  |  |  |
| 13 | Jag märker att jag fungerar betydligt sämre socialt, i arbetslivet eller inom andra viktiga områden (t.ex ansvar för hushållet) till följd av hans/hennes död |  |  |  |  |  |
| Var god vänd 🡪 | | | | | | |
|  | **Påstående** | **Aldrig** | **Sällan** | **Ibland** | **Ofta** | **Alltid** |
| 14 | Jag har påträngande tankar och bilder kopplade till omständigheterna kring hans/hennes död |  |  |  |  |  |
| 15 | Jag har svårt att tänka på positiva minnen om den döda personen |  |  |  |  |  |
| 16 | Jag har negativa tankar om mig själv i förhållande till förlusten (t.ex. tankar om min egen skuld) |  |  |  |  |  |
| 17 | Jag önskar att få dö för att få vara med den döda personen |  |  |  |  |  |
| 18 | Jag känner mig ensam eller känt avstånd till andra personer |  |  |  |  |  |
| 19 | Det känns overkligt att han/hon är död |  |  |  |  |  |
| 20 | Jag lägger mycket skuld på andra på grund av hans/hennes död |  |  |  |  |  |
| 21 | Det känns som om en del av mig dog med honom/henne |  |  |  |  |  |
| 22 | Det är svårt för mig att uppleva positiva känslor |  |  |  |  |  |
| **Bakgrundsfrågor (**Sätt kryss i rutan) | | | | | | |
| 23 | Jag identifierar mig som | □ Man | □ Kvinna | □ Vill inte uppge | | |
| 24 | Hur gammal är du? | år | | | | |
| 25 | Förvärvsarbetar du? | □ Ja | □ Nej jag är | | | |
| 26 | Vilken är din högsta utbildningsnivå? |  | | | | |
| 27 | Hur länge sedan är det din närstående dog? |  | | | | |
| 28 | Vilken relation hade du till din närstående? | Jag var | | | | |
| 29 | Fick du någon form av efterlevandestöd | □ Jag fick | | | | □ Nej |
| 30 | Om du har fått efterlevandestöd, beskriv hur det har hjälpt dig i ditt sorgearbete. | | | | | |
